# Emission of five OAM dispersive waves in dispersion-engineered double-ring core fiber

**DOI:** 10.1038/s41598-024-57587-w

**Published:** 2024-04-11

**Authors:** Wenpu Geng, Yuxi Fang, Changjing Bao, Zhongqi Pan, Yang Yue

**Affiliations:** 1 Institute of Modern Optics, Nankai University, Tianjin, 300350 China; 2 Department of Electrical Engineering, University of Southern California, Los Angeles, CA 90089 USA; 3 Department of Electrical & Computer Engineering, University of Louisiana at Lafayette, Lafayette, LA 70504 USA; 4 School of Information and Communications Engineering, Xi’an Jiaotong University, Xi’an, 710049 China

**Keywords:** Fibre optics and optical communications, Supercontinuum generation

## Abstract

Beams carrying orbital angular momentum (OAM) have exhibited significant potential across various fields, such as metrology, image coding, and optical communications. High-performance broadband coherent OAM sources are critical to the operation of optical systems. The emission of dispersive waves facilitates the efficient transfer of energy to distant spectral domains while preserving the coherence among the generated frequency components. Light sources that maintain consistency over a wide range can increase the efficiency of optical communication systems and improve the measurement accuracy in imaging and metrology. In this work, we propose a germanium-doped double ring-core fiber for five OAM dispersive waves (DWs) generation. The OAM_1,1_ mode supported in the fiber exhibits three zero-dispersion wavelengths (ZDWs) located at 1275, 1720 and 2325 nm. When pumped under normal dispersion, the output spectrum undergoes broadening and exhibits five DWs, situated around 955, 1120, 1450, 2795 and 2965 nm, respectively. Concomitant with blue-shifted and red-shifted dispersive waves, the spectrum spans from 895 to 3050 nm with high coherence. The effect of the fiber and input pulse parameters on DWs generation, as well as the underlying dynamics of the dispersive wave generation process, are discussed. As expected, the number and location of DWs generated in the output spectrum have agreement with the prediction of the phase-matching condition. Overall, this multiple DWs generation method in the proposed fiber paves the way for developing efficient and coherent OAM light sources in fiber-based optical systems.

## Introduction

Short pulses can undergo substantial spectral broadening while propagating throughout nonlinear media, resulting from intricate interactions of nonlinear mechanisms. This phenomenon is known as supercontinuum (SC) generation, and it relies on diverse mechanisms to produce rich phenomena. Over decades of research and development, SC has found applications in optical coherence tomography^1–3^, precision metrology^4^, and molecular spectroscopy^5,6^. Additionally, SC spectra can serve as coherent broadband light sources in optical communications systems^7,8^. Recent research indicate that orbital angular momentum (OAM) beams with the unique phase distribution^9,10^, introducing new degrees of freedom for space division multiplexing^11,12^, adhere to the principles of traditional nonlinear optics^13–15^. With a large effective refractive index spacing between adjacent OAM modes across the spectra, the output mode could maintain the same polarization and state as the input pump pulse. Consequently, supercontinuum generation (SCG) emerges as a promising alternative for achieving coherent broadband OAM light sources in lieu of free-space optical devices.

Dispersive waves (DWs), generated through spectral resonances, play a pivotal role in exciting and enhancing nonlinear mechanisms, facilitating efficient frequency conversion, and promoting spectral broadening within supercontinuum source^16,17^. DWs have potential applications in diverse research fields, encompassing medical diagnostics^18^, optical frequency combs^19^, and bio-photonics^20^. Beyond the realm of nonlinearity, dispersion assumes a paramount significance throughout the entire pulse transmission process. The sign of dispersion at the wavelength of the incident pulse is a decisive parameter that dictates the intricate dynamics encountered during the pulse transmission^21–24^. Previous research on dispersive wave (DW) emission has primarily been associated with soliton fission, a phenomenon typically induced by pumping in an anomalous dispersion regime^25–28^. However, recent investigations have revealed that dispersive waves can also be emitted when the input pulse is pumped under normal group velocity dispersion (GVD). Due to strong self-phase modulation (SPM), the input pulse can extend from normal dispersion to anomalous dispersion, forming solitons that emit dispersive waves in the normal dispersion region^29,30^. Furthermore, non-soliton pulses propagating under normal dispersion condition could emit DWs in the anomalous dispersion region, facilitated by the phase matching between the shock front generated by optical wave breaking (OWB) and the resonant DW^31–33^. In comparison to pumping under anomalous dispersion, the utilization of normal dispersion demonstrates heightened resilience to noise, consequently yielding an output spectrum characterized by exceptional coherence^34–36^. Research about multiple DWs generation of OAM modes is currently in the infancy. R. Scheibinger et al*.* achieved dual dispersive wave generation for high-order modes in normal dispersion when pumping a liquid-core fiber under anomalous dispersion regime^37,38^. Sharma et al. realized SCG of vortex beams accompanied by DW generation by pumping photonic crystal fiber in the normal dispersion region^39^. The transfer of the spectral energy to anomalous dispersion region contributed to the DW generation. The number of generated dispersive waves is limited, and the mechanisms behind the generation of multiple DWs have not been extensively studied.

To primarily focus on the study of OAM DWs generation and provide a comprehensive explanation of the corresponding underlying mechanism, we present a germanium-doped optical fiber in which multiple OAM DWs emitted in both anomalous and normal dispersion regions. The dual-ring core structure provides enhanced flexibility in dispersion control, thereby refining the phase-matching condition. When the input pulse is pumped in the normal dispersion, the resulting spectrum undergoes broadening from 895 to 3050 nm at – 40 dB level and manifests five distinct peaks, situated around 955, 1120, 1450, 2795 and 2965 nm, respectively. The spectrum across the entire bandwidth maintains a high degree of coherence, even for DWs separated from the main body of the spectrum. The influence of the structure and material on DW generation are discussed. This proposed fiber design facilitates the development of a broadband, coherent OAM source, catering to applications in optical coherence tomography, coherent control, metrology, and spectroscopy.

## Results

### Concept and mode property

**Figure 1:**
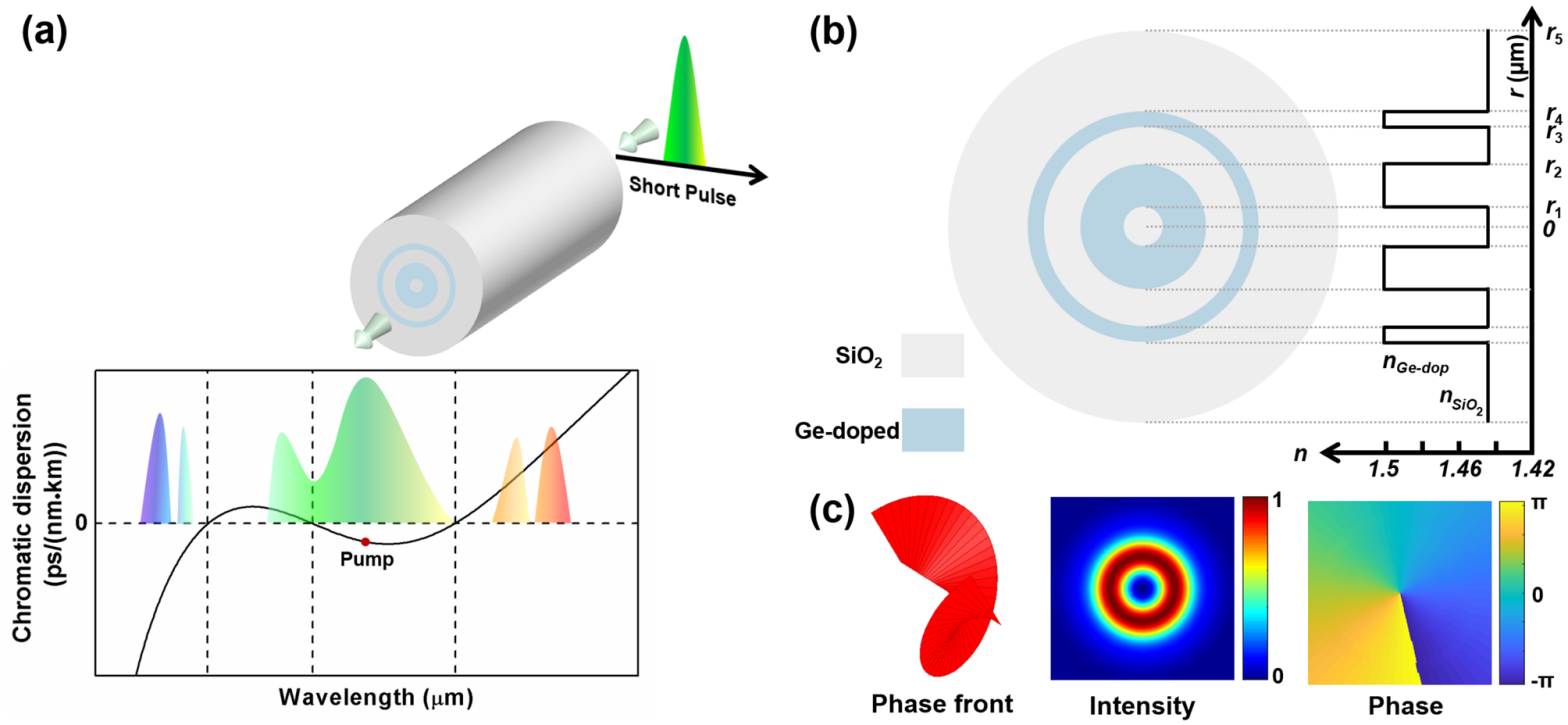
(**a**) Schematic diagram of the five DWs generation process in the designed fiber. (**b**) Cross section and refractive index distribution of the double-ring core optical fiber and corresponding characteristic parameters. (**c**) Helical phase fronts, intensity and phase distributions in the beam cross section for the OAM_1,1_ mode.

Owing to the absence of diffraction effects and strict optical limitations, optical fibers have proven highly efficient for spectral broadening and the generation of DWs^38,40^. The ring-core optical fiber, in particular, offers enhanced freedom in dispersion engineering and serves as an ideal medium for the stable transmission of OAM modes. The schematic of five DWs generation process for the OAM_1,1_ mode in the proposed double-ring-core fiber is depicted in Fig. 1a. The chromatic dispersion (CD) profile of five DWs generation, containing two each of normal and anomalous dispersion regions, is also indicated. The input pulse is pumped in the normal GVD regime. Two DWs are emitted in the normal dispersion regime, and three DWs are formed in the anomalous dispersion regime. In Fig. 1b, the cross section and refractive index profile of the proposed fiber at 1550 nm are illustrated, featuring two 40 mol% Ge-doped rings (*n*_Ge-dop_ = 1.503) surrounded by a cladding of pure silica (*n*_SiO2_ = 1.444). The material refractive indices with wavelength involved in the simulation are calculated by the Sellmeier equations^41,42^. The fiber's modifiable doping concentration, combined with its variable structural parameters, plays a pivotal role in enabling the agile manipulation of dispersion characteristics. The ring-shaped intensity and helical phase distributions of the OAM_1,1_ mode, generated by the linear combination of the even and odd HE_2,1_ modes, are depicted in Fig. 1c.

**Figure 2:**
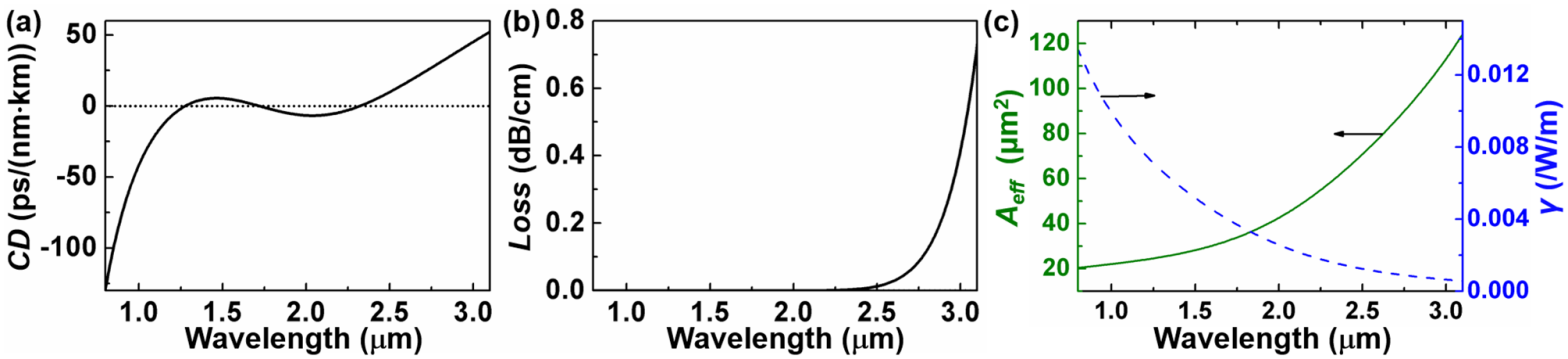
(**a**) CD, (**b**) loss, (**c**) effective mode area and nonlinear coefficient of OAM_1,1_ mode in the optimized fiber.

Properties of the OAM mode supported in the proposed fiber are analyzed by the finite element method (FEM). Figure 2a displays the dispersion property *CD* of the OAM_1,1_ mode as a function of wavelength in the proposed fiber with optimized parameters: *r*_1_ = 1 μm, *r*_2_ = 2.9 μm, *r*_3_ = 4.2 μm, *r*_4_ = 4.8 μm and *r*_5_ = 62.5 μm. The OAM_1,1_ mode exhibits three zero-dispersion wavelengths (ZDWs) located at 1275, 1720 and 2325 nm, covering two regions of normal dispersion and two regions of anomalous dispersion. Although losses are generally ignored since supercontinuum generation with femtosecond pulse pumping occurs in millimeter or centimeter fibers, this analysis considers material losses to distinguish between fibers with varying doping concentrations. The attenuation of OAM modes propagated within this fiber is calculated by taking the loss of two materials into the imaginary component of the material refractive index used in FEM^43,44^. The loss of the mode increases with wavelength, reaching 0.42 dB/cm at 3 µm as depicted in Fig. 2b. Figure 2c depicts the effective mode area *A*_eff_ and nonlinear coefficient *γ* of OAM_1,1_ in the designed fiber. *γ* is calculated using a full vector model^45^, considering the effective mode area *A*_eff_ and the nonlinear refractive indices *n*_2_ in different regions. In this fiber, $n_{2} = 2.16 + 0.033M$ with the unit 10^−20^ m^2^/W^46,47^, where *M* is the Ge-doped mole fraction. As the effective mode area increases with the wavelength, the nonlinear coefficient decreases. At 1.97 µm, the effective mode area is 41.38 µm^2^, and the corresponding nonlinear coefficient *γ* is 0.0027 /W/m.

### DWs engineering and methods

Dispersive wave must be propagated with the same phase velocity as that of the nonlinear pump^33,48^. When calculating the radiated frequency of the input pulse under either anomalous dispersion or normal dispersion pumping conditions, the phase-matching condition has been demonstrated to possess the capability to predict the position and number of DWs in the output spectrum as follows

1 $$\beta {(}\omega {)} = \beta {(}\omega_{s} {)} + {(}\omega - \omega_{s} {)(}\frac{d\beta }{{d\omega }}{)}_{{\omega_{s} }} + C$$

where *ω* and *ω*_s_ denote the frequencies of DWs and soliton respectively, *β*(*ω*) and *β*(*ω*_*s*_) are the corresponding propagation constants, *C* represents the constant term *γP*_s_/2, which is related to the Kerr nonlinear coefficient *γ* and soliton peak power *P*_s_. This term, contingent on the power variations, embodies the nonlinear phase-shift and is typically neglectable compared to the other terms in Eq. (1). However, in situations where the equation is responsive to the nonlinear contribution, due consideration of this constant term becomes necessary. The accurate assessment of the soliton′s peak power is challenging due to the pulse distortion caused by dispersion and nonlinear effects during propagation. Therefore, obtaining a solution to Eq. (1) is difficult. To simplify the calculations and aid the visualization, this condition is generally adjusted to the phase mismatch between the DW and the soliton and expanded via Taylor series as

2 $$\Delta \beta \left( \omega \right) = \beta \left( \omega \right) - \beta \left( {\omega_{s} } \right) - \left( {\omega - \omega_{s} } \right)\left( {\frac{d\beta }{{d\omega }}} \right)_{{\omega_{s} }} - C = \sum\limits_{n = 2} {\frac{{\left( {\omega - \omega_{s} } \right)}}{n!}}^{n} \frac{{d^{n} }}{{d\omega^{n} }}\beta \left( {\omega_{s} } \right) - C$$

**Figure 3:**
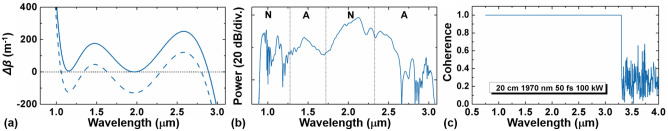
(**a**) Phase mismatch *Δβ*, and (**b**) simulated output spectrum of the proposed fiber with *L* = 20 cm. (**c**) Coherence of the generated spectra.

The Taylor series expansion is used to determine the order of the propagation constant that is brought into the subsequent SC generation formula. Here, the constant term *C* could shift the phase mismatch curve overall downward. Figure 3a illustrates the *Δβ* characteristics of the OAM_1,1_ mode in the optimized fiber when the input short pulse pumped at 1970 nm experiences normal dispersion of − 6.36 ps/(nm·km). The blue solid line indicates the condition in which *P*_s_ is negligible and the blue dashed line represents the condition that *Δβ* is sensitive to the soliton peak power. When considering the effect of nonlinear contributions, the phase mismatch curve potentially has five intersection points with Δ*β* = 0. This indicates the potential to produce five DWs. As depicted in the figure, it is evident that within a specific range, approximately five phase-matching points can be roughly predicted.

To validate this prediction, the corresponding output spectrum in Fig. 3b is simulated by the generalized pulse-propagation equation (GNLSE) as

3 $$\begin{aligned} & \frac{\partial A}{{\partial {\text{z}}}} + \frac{\alpha }{2}A - i\sum\limits_{n = 2}^{\infty } {\frac{{i^{n} \beta_{n} }}{n!}} \frac{{\partial^{n} A}}{{\partial t^{n} }} \hfill \\ & = i\gamma \left( {1 + \frac{i}{{\omega_{0} }}\frac{\partial }{\partial t}} \right) \times \left( {A(z,t)\int_{0}^{\infty } {R(t^{\prime} )} \left| {A(z,t - t^{\prime} )} \right|^{2} dt^{\prime} } \right) \hfill \\ \end{aligned}$$

where *A* = *A*(*z*,*t*) represents the electric field envelope, *α* is the loss, *β*_n_ represents the *n-*th order dispersion coefficient obtained through the Taylor series expansion of the propagation constant *β*(*ω*) about the center frequency *ω*_0_, and *t*′ is the present time frame. In the simulation, the GNLSE is solved by the split-step Fourier method. The parameters of the incident Gaussian pulse are listed in Table 1, where *P* represents the peak power, *λ*_0_ is the center wavelength, *t*_fWHM_ is the full width half maximum, and *L* is the length of fiber. The resulting spectrum undergoes broadening from 895 to 3050 nm at − 40 dB level and exhibits five distinct peaks, situated around 955, 1120, 1450, 2795 and 2965 nm, respectively. The positions of these five DWs roughly coincide with the *Δβ* = 0 points of the dotted line in Fig. 3a. The relatively complex phase mismatch also demonstrates the possibility of multiple dispersive wave generation at other pump wavelengths. The steep rise in dispersion and loss at longer wavelengths restricts the expansion of supercontinuum. However, by adjusting the fiber parameters, further broadening of the output spectrum can be achieved. In practical experiments, the fiber laser system built by G. Prabhakar and colleagues serves as a reference^14^. Furthermore, they perform detection and purity measurements of OAM beams to verify the state of the output modes.

**Table 1:** Fiber structural parameters, input pulse characteristics, and DW positions under different fiber doping concentrations (* denotes the same parameters).

*M*	*r*_1_ (μm)	*r*_2_ (μm)	*r*_*3*_ (μm)	*r*_4_ (μm)	*P* (kW)	*λ*_0_ (nm)	*L* (cm)	DW_1_ (nm)	DW_2_ (nm)	DW_3_ (nm)	DW_4_ (nm)	DW_5_ (nm)
20 mol%	1	4	6.5	8	200	1870	30	920	1070	1385	2175	2600
40 mol%	1	2.9	4.2	4.8	100	1970	20	955	1120	1450	2795	2965
60 mol%	1	2.8	4	4.8	125	2010	20	930	1105	1575	3200	3340

^*^r_5_ = 62.5 μm, *t*_FWHM_ =50 fs

To assess the coherence of this spectrum, quantum noise is introduced to the input pulse, and the coherence is calculated by conducting the simulation 40 times. The degree of the first-order coherence is determined as^48^:

4 $$g_{12} \left( \omega \right) = \frac{{\left\langle {\left. {\tilde{A}^{*}_{1} \left( {L,\omega } \right)\tilde{A}_{2} \left( {L,\omega } \right)} \right\rangle } \right.}}{{\left[ {\left\langle {\left. {\left| {\tilde{A}_{1} } \right.\left( {L,\omega } \right)} \right|^{2} } \right\rangle \left\langle {\left. {\left| {\tilde{A}_{2} } \right.\left( {L,\omega } \right)} \right|^{2} } \right\rangle } \right]^{1/2} }}$$

where $\tilde{A}_{1}$ and $\tilde{A}_{2}$ represents the Fourier transforms of two neighboring pulses and the anglebrackets denote an average over the entire ensemble of pulses. The illustration in Fig. 3c highlights the capacity of this design to maintain a high level of coherence across the entire output spectrum.

## Discussion

### Pulse evolution

**Figure 4:**
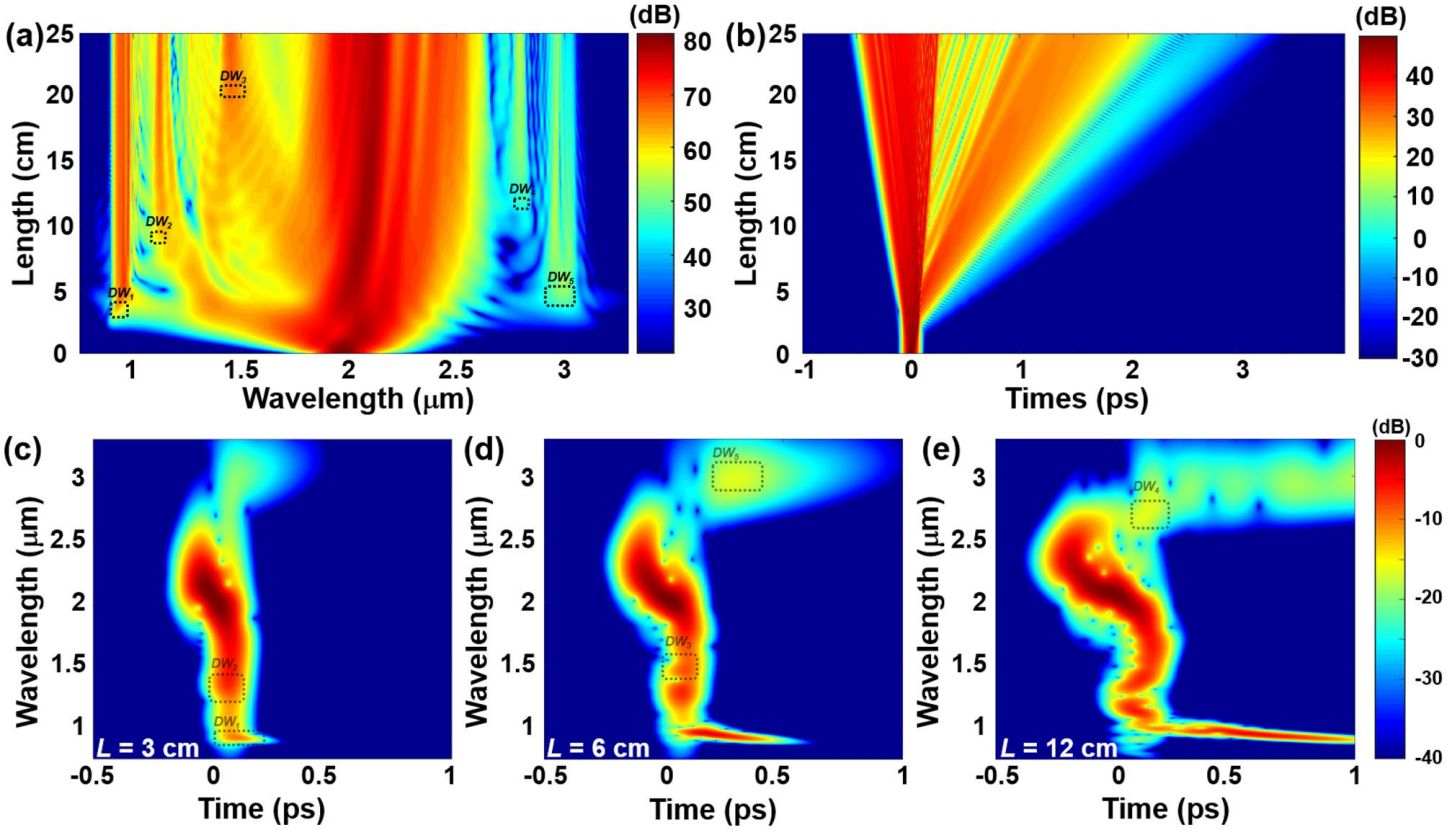
(**a**) Spectral and (**b**) temporal evolutions of OAM_1,1_ mode with five DWs generation in the fiber as a function of propagation distance. Mixed spectral-temporal representation of the optical pulse in the proposed fiber with propagation length (**c**) *L* = 3 cm, (**d**) *L* = 6 cm, (**e**) *L* = 12 cm.

Figure 4a exhibits the evolution of pulse propagation along the designed fiber. To get an intuitive understanding of the phenomena occurring during the pulse propagation, the cross-correlation frequency-resolved optical gating (XFROG) diagrams of the signal propagation is illustrated in Fig. 4c-e, which simultaneously exhibits temporal and frequency evolutions of the spectra. Initially, the spectrum undergoes broadening owing to SPM in the normal dispersion region. Subsequently, the shorter wavelength portion of the spectrum gradually extends into the anomalous dispersion regime. In this region, solitons are generated and later break into fundamental solitons, accompanied by the emission of dispersive wave into normal dispersion domains as shown in Fig. 4c^29,30^. This mechanism explains the successive emission of DW_1_ and DW_2_ in normal dispersion. In nonlinear fibers with weak normal dispersion, pulses undergo wave breaking, stimulating linear dispersive waves in the anomalous dispersion region that are phase-matched with the shock-wave front^32^. The resonant amplification of DWs in the anomalous dispersion regime (DW_3_, DW_4_ and DW_5_) as depicted in Fig. 4d,e can be attributed to this phenomenon, which can be observed at the oscillating pulse front in Fig. 4b^32,49^. After propagating for 20 cm, the output spectrum tends toward stabilization. Therefore, *L* = 20 cm is selected as the primary data point for analysis.

### DWs generation with varied input pulse parameters

**Figure 5:**
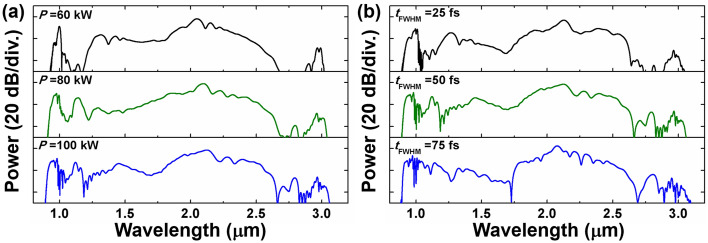
Influence of different input pulse parameters on DWs generation of OAM_1,1_ after the 20 cm long fiber for different (**a**) peak power *P* and (**b**) pulse FWHM *t*_*FWHM*_.

By varying the peak power and duration of the input pulse, the influence of the pump pulse properties on the DWs generation is investigated. Figure 5a illustrates the effects of the peak power *P* on the supercontinuum after 20 cm fiber when *t*_*FWHM*_ is set to 50 fs. With the increment of the input pulse peak power, the nonlinear effects during the pulse propagation are accelerated and intensified, enabling the observation of five DWs generation at *P* = 100 kW. Figure 5b explores the variation of* t*_*FWHM*_ from 25 to 75 fs, with *P* fixed at 100 kW. The increase in the* t*_*FWHM*_ of the input pulse results in a deterioration in the flatness of the output spectra. When maintaining a constant peak power, the input pulse with larger *t*_*FWHM*_ carries more energy and exhibits narrower spectra, which means each frequency component carrying more energy, thereby intensifying the nonlinear effects^50^.

### DWs generation with varied fiber parameters

**Figure 6:**
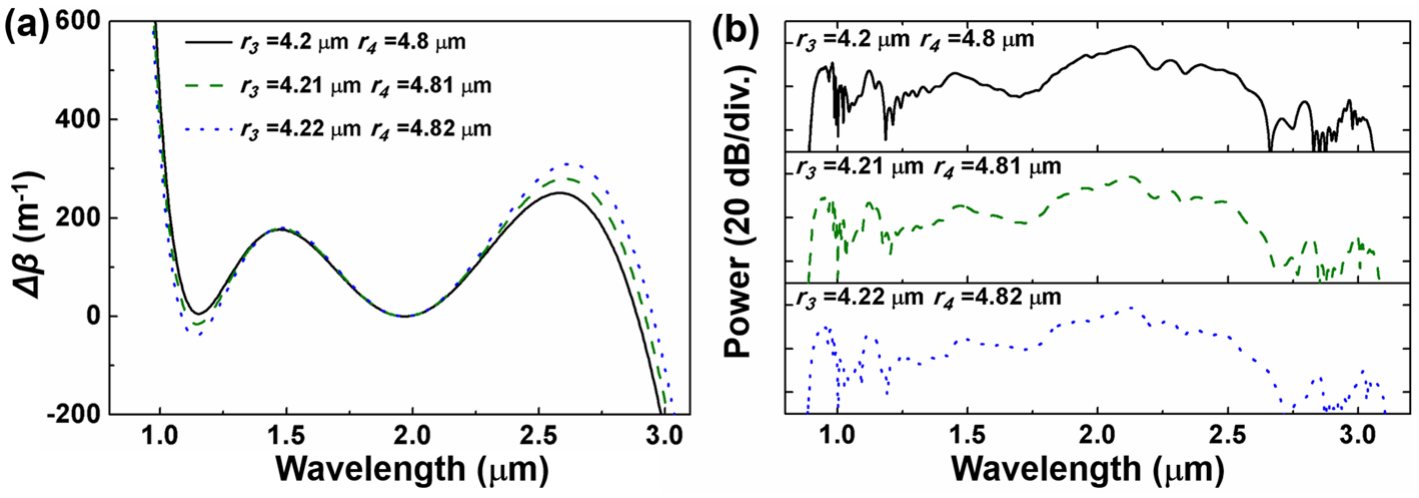
(**a**) Phase mismatch *Δβ* and (**b**) spectra with five DWs generation of OAM_1,1_ mode under different outer ring positions.

To explore the influence of fiber parameters on phase-matching points and to validate the prediction of dispersive waves, the parameter of the proposed fiber is adjusted. The consistency between *Δβ* = 0 points and dispersive wave locations in the output spectrum is confirmed through GNLSE simulations. Figure 6 illustrates the phase mismatch and calculated spectrum with the generation of five DWs for the OAM_1,1_ mode in the proposed fiber, considering different positions of the outer ring. Maintaining *r*_1_ = 1 μm, *r*_2_ = 2.9 μm, *r*_5_ = 62.5 μm and the width of the outer ring 0.6 μm, the position of the outer ring is adjusted by 0.02 μm. The reason for such fine tuning is that the phase mismatch curve is highly sensitive to variations in the fiber parameters. When the parameter adjustment is larger, the phase matching curve no longer has the potential for five zero points, indicating that the proposed fiber has relatively high manufacturing process requirements. Due to the rapid decline of the phase mismatch curve at shorter wavelengths, this alteration exerts a comparatively minor impact in this spectral range. The *Δβ* = 0 point in the long wavelength region experiences a redshift. Consequently, the corresponding position of DW_5_ in Fig. 6 shifts away from the pump wavelength, from 2965 to 3022 nm.

**Figure 7:**
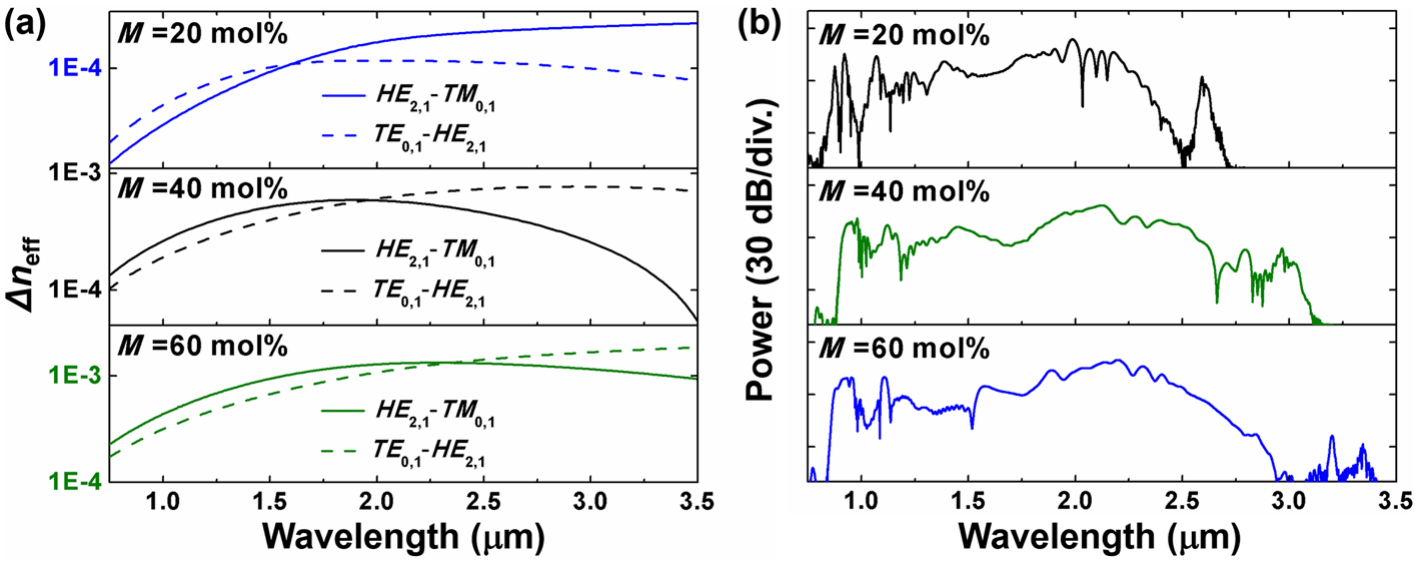
(**a**) Effective refractive index difference between adjacent eigenmodes of the HE_2,1_ mode under different mole fraction, and (**b**) the corresponding spectrum.

Aside from enhancing the linear and nonlinear refractive indices, the variable doping concentration of GeO_2_ allows for more adaptable adjustments to fiber dispersion and output spectral characteristics. Due to the different coefficients of thermal expansion between silica cladding and high refractive index regions, fibers with lower Ge-doped levels are easier to be manufactured^51^ and exhibit lower material losses^52,53^. However, fibers with higher doping levels have a larger refractive index contrast between the high refractive index regions and the cladding, enhancing the confinement of lights within the core. The effective separation between adjacent eigenmodes aids in maintaining the stability of OAM modes. Hence, we investigate the effect of Ge-doped concentration on mode properties supported in the designed fiber. Corresponding to the optimized parameters under different mole fractions and the incident pulse parameters in Table 1, Fig. 7 displays the effective refractive index difference between adjacent eigenmodes of the HE_2,1_ mode and the output spectrum. As shown in Fig. 7a, fibers doped with 40 and 60 mol% exhibit an effective refractive index difference greater than 10^−4^ across the entire work spectra, indicating that the transmitted modes can retain the same mode polarization and state in the output spectrum as the input pump pulse^14,54^. Simultaneously, at the same fiber parameters, the same mode in lower doped fibers could be cut off at shorter wavelengths. To achieve a broad working window for multiple dispersive waves, 20 mol% doped fibers employ larger ring widths to confine the modes. However, the inevitably rapid dispersion increase at longer wavelengths still limits the spectral broadening and, consequently, the positioning of the dispersive waves. As depicted in Fig. 7b, the spectrum significantly broadens at longer wavelengths as the mole fraction increases in steps of 20 mol% up to 60 mol%. Table 1 summarizes the five DWs generated in different doped fibers. The selection of GeO_2_ mole fraction can be customized according to specific applications and practical considerations in fiber manufacturing, allowing for fine-tuning to optimize the performance based on the characteristics of the target scenario.

## Conclusion and prospect

A dispersion-engineered fiber with two Ge-doped ring cores is proposed for generating five coherent OAM DWs. Featuring three ZDWs, this designed contributes to complicated nonlinear effects. When pumped in the normal dispersion regime, five DWs are generated during pulse transmission due to the phase matching with the dispersive shock waves or solitons. Numerical verification confirms a high level of coherence across the entire spectrum bandwidth, containing multiple discrete DWs. The influence of the mole fraction on the effective refractive index difference and output spectra is discussed. This methodology paves the way for creating an efficient OAM supercontinuum source distinguished by a heightened level of coherence.

The first-order OAM mode serves as a baseline for investigation. Meanwhile, this proposed fiber structure has the potential to support higher-order OAM modes with similar dispersion characteristics for DW generation. This design can function as a light source to provide diverse frequency components for super-resolution microscopy^55^. In addition, typical utilizations of OAM modes span an array of domains, from image coding and pattern recognition^56^ to quantum communication^57^ and detection^58^, all requiring OAM mode under target frequency. Our design caters to these needs by offering a broad and high-coherence light source alternatives. However, the designed fiber has high manufacturing precision requirement and doping concentrations is higher than those of currently commercialized fibers. Potential preparation methods including plasma chemical vapor deposition (PCVD)^59^ and the extrusion of glass discs^60^ could be feasible. Furthermore, the mode coupling and stability of the fiber are subject to environmental and mechanical strains, necessitating consideration of compensation mechanisms during fiber drawing and practical application. With the trend towards equipment miniaturization, the optical transmission medium based on this principle could be extended to other structures and materials, potentially involving different dispersion properties and nonlinear effects. Higher-order OAM modes with DW generation, as carriers for mode division multiplexing, also merit further study.

## Data Availability

The datasets used and/or analysed during the current study available from the corresponding author on reasonable request.
